# Monitoring and accountability for the Pacific response to the non-communicable diseases crisis

**DOI:** 10.1186/s12889-016-3614-8

**Published:** 2016-09-10

**Authors:** Hilary Tolley, Wendy Snowdon, Jillian Wate, A. Mark Durand, Paula Vivili, Judith McCool, Rachel Novotny, Ofa Dewes, Damian Hoy, Colin Bell, Nicola Richards, Boyd Swinburn

**Affiliations:** 1 Department of Epidemiology and Biostatistics, School of Population Health, University of Auckland, Private Bag 92019, Auckland, 1142 New Zealand; 2 World Health Organisation, Western Pacific Regional Office, Suva, Fiji; 3 Pacific Research Centre for the Prevention of Obesity and Non-Communicable Diseases (C-POND), Fiji National University, Suva, Fiji; 4 Pacific Islands Health Officers Association, Honolulu, HI USA; 5 Pacific Community, Noumea, New Caledonia; 6 University of Hawaii, Honolulu, HI USA; 7 School of Population Health & Department of Molecular Medicine, University of Auckland, Auckland, New Zealand; 8 School of Medicine, Deakin University, Geelong, Australia; 9 School of Population and Global Health, University of Melbourne, Melbourne, Australia

**Keywords:** Non communicable diseases, Monitoring and accountability, Dashboard, Pacific, Policy

## Abstract

**Background:**

Non-communicable diseases (NCD) are the leading cause of premature death and disability in the Pacific. In 2011, Pacific Forum Leaders declared “a human, social and economic crisis” due to the significant and growing burden of NCDs in the region. In 2013, Pacific Health Ministers’ commitment to ‘whole of government’ strategy prompted calls for the development of a robust, sustainable, collaborative NCD monitoring and accountability system to track, review and propose remedial action to ensure progress towards the NCD goals and targets. The purpose of this paper is to describe a regional, collaborative framework for coordination, innovation and application of NCD monitoring activities at scale, and to show how they can strengthen accountability for action on NCDs in the Pacific. A key component is the Dashboard for NCD Action which aims to strengthen mutual accountability by demonstrating national and regional progress towards agreed NCD policies and actions.

**Discussion:**

The framework for the Pacific Monitoring Alliance for NCD Action (MANA) draws together core country-level components of NCD monitoring data (mortality, morbidity, risk factors, health system responses, environments, and policies) and identifies key cross-cutting issues for strengthening national and regional monitoring systems. These include: capacity building; a regional knowledge exchange hub; innovations (monitoring childhood obesity and food environments); and a robust regional accountability system.

The MANA framework is governed by the Heads of Health and operationalised by a multi-agency technical Coordination Team. Alliance membership is voluntary and non-conditional, and aims to support the 22 Pacific Island countries and territories to improve the quality of NCD monitoring data across the region. In establishing a common vision for NCD monitoring, the framework combines data collected under the WHO Global Framework for NCDs with a set of action-orientated indicators captured in a NCD Dashboard for Action.

**Summary:**

Viewing NCD monitoring as a multi-component system and providing a robust, transparent mutual accountability mechanism helps align agendas, roles and responsibilities of countries and support organisations. The dashboard provides a succinct communication tool for reporting progress on implementation of agreed policies and actions and its flexible methodology can be easily expanded, or adapted for other regions.

## Main text

### Background

Non-communicable diseases (NCD), principally cardiovascular diseases, cancer, diabetes and chronic respiratory diseases, have become the leading cause of premature death and disability in the Pacific region [1, 2]. In 2011 Pacific Islands Forum leaders and ministers of health declared the Pacific region to be in “a human, social and economic crisis” due to the significant and growing burden of NCDs [3–5]. The prevalence of NCD risk factors (high obesity, tobacco use, alcohol abuse, elevated fasting blood glucose and hypertension) and the ensuing social and economic impact of premature mortality, morbidity, lost productivity, and escalating health care expenditure [2] poses one of the biggest threats to development across the region [6]. Recent studies show that twelve countries with highest diabetes prevalence 1 and obesity prevalence 2 are Pacific Islands countries or territories (PICTs) 3 [7, 8].

As noted by Gouda and colleagues [9], post millennium development goal debates have shifted from a ‘what works’ approach to issues of accountability - ‘ensuring what has been agreed gets done’ – and monitoring systems are essential to achieving this. In keeping with this shift, in 2013 there was regional ministerial commitment to develop a cost-effective, coordinated, ‘whole of government’ strategy, aimed at identifying priority areas and high impact policy actions [10, 11], and for the development of “a regional and national NCD accountability mechanism to monitor, review and propose remedial action to ensure progress towards the NCD goals and targets” [12]. Several outcomes have emerged from these commitments: (i) an overarching Pacific NCD Roadmap [13] that highlights a data-driven, evidential approach and emphasises commitments for greater collaboration and resources to tackle the NCD crisis [10]; (ii) the development of nationally relevant NCD goals and targets that align with the global goals (e.g. World Health Organisation Global Monitoring Framework (GMF) and the Global Action Plan for the prevention and control of NCDs, 2013-2020) [14]; and (iii) the establishment of the Monitoring Alliance for NCD Action (MANA) for effective monitoring of a complex set of NCDs and their risk factors. The Pacific faces a number of challenges that necessitate collaboration, innovation, scale and accountability in its response to NCDs. The MANA is one of the ways in which partners are endeavouring to work together to derive and implement this response.

The purpose of this paper is to describe the development of a regional, collaborative framework for coordination, innovation and application of NCD monitoring activities at scale, and to show how they can strengthen accountability for action on NCDs in the Pacific.

### The context for pacific NCD monitoring and action

Since the early 2000s, high- level political support for addressing NCDs has been strong with ministerial endorsement for a plethora of global and regional commitments 4. In 2007, the region embarked on the ambitious five-year *Pacific Regional 2-1-22 Non-Communicable Disease Program (2007-2011)*^5^ under which many PICTs developed, costed and prioritised strategies aimed at NCD reduction. By the end of the initiative, although NCD monitoring and capacity had increased considerably (and continues to increase), routine NCD monitoring systems in most countries were still underdeveloped [15]. 2011, however, represents a watershed. Deeply concerned by the growing economic and social burdens caused by NCDs, the Pacific Forum leaders declared an NCD crisis for the region and reiterated calls for a more systematic, collective approach to tackle it.

Post Declaration progress in NCD monitoring has been significant, with considerable growth in a number of areas. Three examples include: (i) a rise in the number of epidemiology technicians equipped to conduct NCD monitoring activities. This has been due to Pacific Public Health Surveillance Network (PPHSN)’s newly implemented training and capacity development programme for ‘Strengthening Health Interventions in the Pacific (SHIP)’ which includes several Data for Decision-Making training modules, and the development of an integrated approach to NCD monitoring and policy intervention in the northern Pacific, led by the Pacific Islands Health Officers’ Association (PIHOA). (ii) Since 2002 most PICTs (although not all) have undertaken at least one national population survey using the WHO STEPwise (STEPS) risk factor approach to NCD control (or equivalent). These stimulate action from the first survey, and convey progress by tracking trends across subsequent surveys. However, regular risk factor surveys are not yet routine, with only nine countries having completed two, which limits comparability across the region. (iii) Civil registration and vital statistics (CRVS) and health information systems are critical for accurately determining cause of death and these systems continue to improve. The Pacific Vital Statistics Action Plan (2011-2014), implemented by the Brisbane Accord Group, was designed to assist countries improve collection and make better use of mortality data, including the measurement of NCDs [16, 17]. This extensive plan is now into its second phase (2015-20) and is a key component of the Ten Year Pacific Statistics Strategy (2011-2020).

Notwithstanding these efforts, ongoing improvement is needed to enhance existing monitoring efforts to a level that can reliably inform policy actions to tackle the NCD crisis. Further, due to the number of efforts being undertaken, harmonisation, coordination and closer collaboration are critical priorities to avoid the negative impacts of fragmentation on PICT health systems.

## Discussion

### The pacific monitoring alliance for NCD action (MANA)

MANA was conceived as a sustainable collaborative platform for NCD monitoring and accountability with a three-pronged strategic approach: (i) *To support in-country capacity* to identify and understand national NCD monitoring strengths and weaknesses, and raise awareness of services available to address their prioritised needs. (ii) *To support growth of Regional Public Goods* - technical expertise and regional services - to build national and regional technical data capacity and knowledge exchange to effectively monitor NCDs; and (iii) *To support monitoring innovation and develop mutual accountability systems.* The innovation component includes promoting important new or currently under-resourced NCD monitoring areas such as monitoring food environments and childhood obesity trends. Developing mutual accountability mechanisms for national and regional review of NCD actions, with constructive feedback to decision-makers in PICTs and Pacific organisations, requires innovative data collection methods and an independent assessment system for measuring actions to reduce NCDs.

The voluntary alliance has no conditions for membership and serves all 22 PICTs and relevant technical partners active in NCD monitoring, drawing them together to better utilise the extensive NCD data-related activity already underway across the region. MANA technical partners include: the Pacific Community (SPC); the World Health Organisation (WHO); the US Centres for Disease Control and Prevention (CDC); the Pacific Research Centre for Prevention of Obesity and NCDs (C-POND), based at Fiji National University; the Pacific Islands Health Officers’ Association (PIHOA); and several universities.

**Fig. 1:**
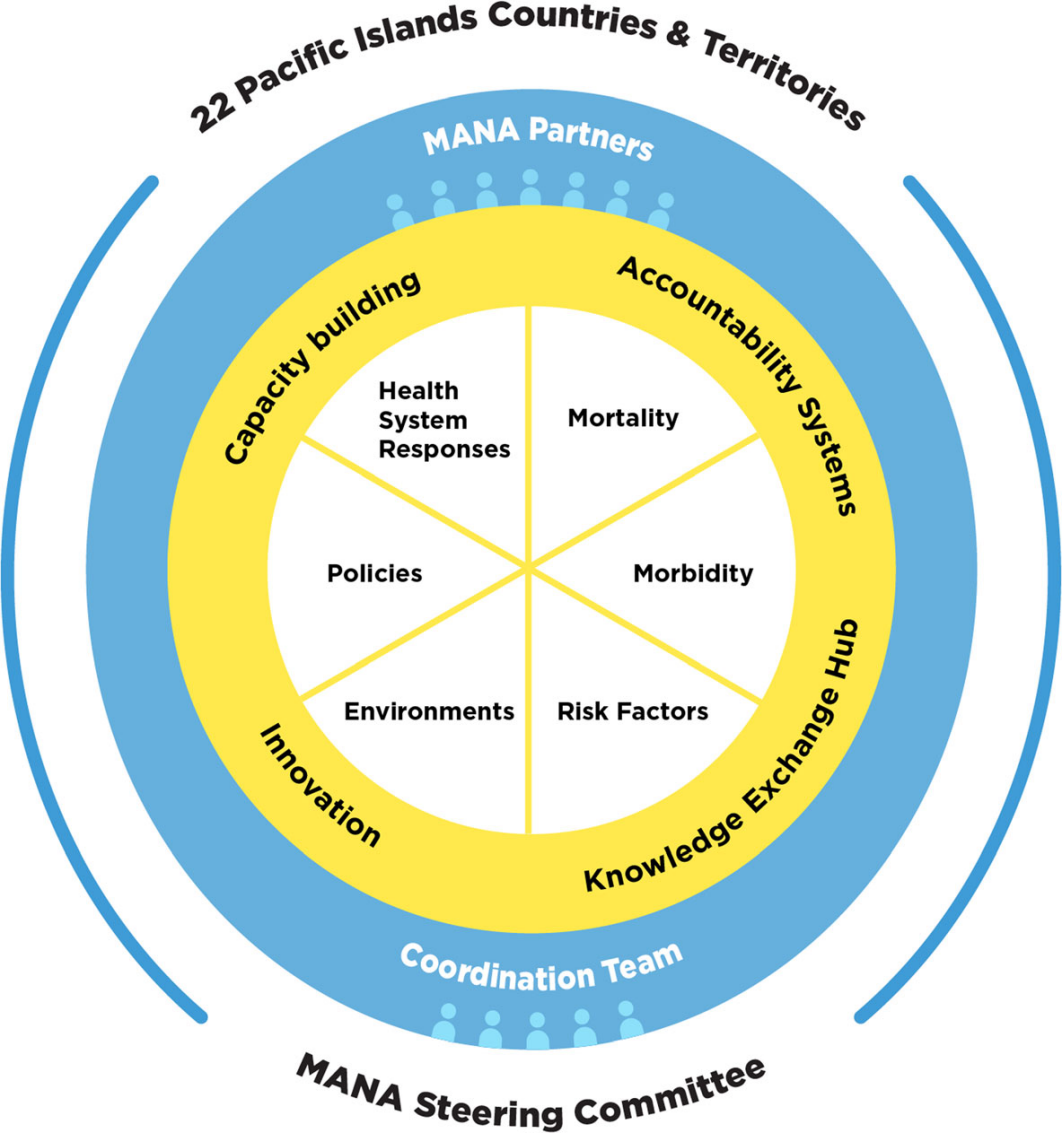
The Pacific Monitoring Alliance for NCD Action (MANA) Framework

A framework emerged from multiple meetings and negotiations and was endorsed by senior Pacific health officials (Fig. 1). It clarifies the main NCD monitoring components and activities of the alliance (yellow) and the governance and coordination mechanisms for the alliance partners (the outer thin and thick blue rings). At its centre are six core NCD monitoring components surrounded by four cross-cutting priorities: ‘*capacity building’, ‘knowledge exchange ‘, ‘innovation’,* and *‘accountability’* [18]. It is hoped that by adopting all six monitoring components, countries will be able to build their minimum datasets [9] and implement a comprehensive monitoring system over time.

While most of the six core components draw upon established tools and protocols (Table 1), the ‘environment’ and ‘policies’ monitoring components were least developed. With funding and technical support under MANA, the C-POND team has adapted INFORMAS 6 protocols for use in the Pacific. In particular, seven Pacific protocols for monitoring the food environment and food policies have been piloted and are now ready for roll-out across the region from 2016. To date, baseline monitoring has been completed in Fiji and several other PICTs, including Cook Islands, Tonga, Tokelau and Nauru, have requested assistance to set up baseline food monitoring systems as soon as possible. The policies component is reinforced by the development of the Pacific MANA Dashboard for Action, a key component of the framework [18]. This multi-layer monitoring and communication tool strengthens mutual accountability by providing a mechanism for governments to demonstrate leadership through targeted policies and legislation aimed at reducing NCDs.

**Table 1:** The six monitoring components and current status

	Monitoring Component	Current Status
Mortality	Age, sex and causes of death are critical for defining the extent of the impact of NCDs on a population and monitoring reductions in probability of dying from NCDs.	• Forms part of the country’s broader, multi-sectoral CRVS.
		• Since 2011 under the 10 yr Pacific Vital Statistics Action Plan, significant progress has been achieved in strengthening PICTs’ CRVS systems and health information systems [16]. Substantial gains in coverage, quality, data use and accessibility have been made; most importantly is growing country commitment and engagement. Ensuring countries can at least report accurate, all-cause mortality by age group is a priority (the 15-59 age group being a close proxy for premature NCD mortality), alongside continuing improvements in cause of death data.
		• The Pacific SHIP Program is working alongside the Brisbane Accord Group initiative to strengthen in-country capacity for monitoring of mortality.
Morbidity	Self-reported diseases, mainly diabetes and cardio-vascular disease.	• Data collection is generally problematic as central disease registries are not common.
		• Self-reported conditions captured by the WHO STEPS survey or CDC Behavioral Risk Factor Surveillance System (BRFSS).
		• The Pacific SHIP Program is working to strengthen in-country capacity for monitoring of NCD morbidity.
Risk Factors	NCD risk factors include tobacco use, harmful use of alcohol, diet, physical inactivity, obesity and hypertension	• STEPS and BRFSS surveys provide the prevalence data.
		• The WHO Global School-based student Health Survey and CDC Youth Risk Factor Behaviour Survey provide data for adolescents.
		• By 2015, 19 PICTs have completed at least one adult and one adolescent NCD risk factor survey [39].
		• North Pacific – South Pacific variation and survey changes over time makes some regional or cross-country comparisons difficult. Some initial research is underway to assess where changes could be made.
		• The Pacific SHIP Program is working to strengthen in-country capacity for monitoring of NCD risk factor prevalence.
Environments	The physical, economic, policy and socio- cultural environments that influence diet, tobacco use, alcohol uptake and physical activity.	• The food environment has been identified as a target for the Pacific.
		• Some environment indicators are included in existing monitoring frameworks (e.g. policies to limit saturated fats and virtually eliminate trans-fats in the WHO GMF; tobacco indicators in the WHO MPOWER measures [40]).
		• The INFORMAS ^6^ group has developed a series of monitoring tools to measure food environment indicators [25]. These are being adapted for the Pacific by researchers at C-POND at Fiji National University.
Policies	Policy indicators are ‘solution’ indicators – they indicate what governments are doing to tackle the NCD crisis.	• The Pacific NCD Roadmap initiative encourages governments to undertake a range of multi-sectoral cost-effective, ‘best buy’ policy directives that will impact legislation [13]. Some key policy data are collected by WHO through Country Capacity Surveys.
		• Some food policy monitoring is included in food environment work being carried out by C-POND.
		• The US Affiliated Pacific Islands NCD Policy Commitment Package is a Pacific-customized, expanded set of set of legislative, regulatory, and institutional policies endorsed by the health Secretaries, Directors and Ministers in the US-affiliated Pacific, which can be incorporated into the MANA dashboard on request [23].
		• Boosted by the INFORMAS^6^ approach and drawing on other existing tools, the development of the Pacific MANA Dashboard for Action will provide a multi-layer monitoring tool and an accountability mechanism for governments to demonstrate leadership through targeted policies and legislation aimed at reducing unhealthy lifestyle choices.
Health System Responses	This covers monitoring of the use and accessibility to essential medicines, cardio-vascular disease risk assessment, drug therapy and counselling.	• For member countries, some data are captured on the WHO Country Capacity Surveys.
		• For countries participating in the regional rollout of the WHO Package of Essential NCD interventions for primary health care, establishing an integrated monitoring system within the NCD plan will be beneficial [41].

### Capacity building

Low levels of capacity in data and epidemiology skills among public health workers in the region limits availability and translation of monitoring data in the Pacific. While a number of workshops delivered in the region over the years have attempted to address this, PPHSN’s newly implemented SHIP programme is the first systematic regional approach to building a workforce of epidemiologists and epi-technicians in the Pacific [19]. By the end of its first phase in 2015, five accredited Data for Decision Making courses, inclusive of communicable and non-communicable diseases, had been delivered through 39 on-site and regional classes to over 250 epi-technician candidates in 16 PICTs.

As part of child obesity monitoring efforts in the north Pacific, the Children’s Healthy Living Program for Remote Underserved Minority Populations in the Pacific Region (CHL) has trained 150 field anthropometrists to collect standardised early childhood data [20, 21]. From 2016 the CHL Summer Institute will broaden the training reach by offering it through an online credit and non-credit (continuing education) programme [22] and has been expanded to include all age groups.

The WHO Pacific Open Learning Health Net (POLHN) has developed a range of high quality, on-line resources related to epidemiology and NCD control. Further resourcing is required to scale-up these initiatives; however, additional efforts at strengthening skills should align with regional initiatives rather than create duplicate mechanisms.

MANA partners will continue to build and deliver capacity building programmes such as those described above. However, the strengthened collaboration and harmonisation that MANA brings will help ensure agencies work to their comparative advantage to improve the quantity and focus of programmes, and ensure capacity gaps are filled to improve overall the monitoring of NCDs.

### Knowledge exchange

A regional knowledge hub is envisaged as a ‘go-to’ platform for partners with the aim of providing ready access to a range of available databases and developing combined/ integrated data resources to enable interactive use. In addition, it would enable users to access information and NCD monitoring related resources and tools; serve as a forum to share ideas, events and courses; and serve as a go-to advice and support portal. A user-friendly, technologically sophisticated platform will be challenging to establish, both technically and collaboratively, but MANA technical partners are committed to making data and information more readily available to all health professionals, policy makers and interested parties in formats that can quickly and effectively inform their NCD actions and decisions.

NCD-related knowledge exchange collaborations are gradually becoming stronger across the region. Research relationships among MANA partners, for example, are evident under the CHL Program (www.chl-pacific.org), which closely links the University of Hawai’i with the United States affiliated Pacific Islands (USAPI) 7. Cross regional (north-south Pacific) research links are also growing, particularly in relation to child BMI monitoring and food monitoring tools, protocols and training, and PIHOA’s NCD Policy toolbox [23, 24].

### Innovation

The innovation component focuses on developing under-developed but important monitoring areas. These currently include monitoring food environments and related policies; monitoring childhood obesity; and developing lower-cost population surveys.

As noted earlier and in Table 1, a number of INFORMAS ^6^ monitoring protocols [25] have been adapted and piloted for the Pacific by researchers at C-POND and are now ready to be used to undertake baseline assessments across the region. The piloted protocols include food composition, food labelling, food nutrient content, food promotion in schools, food advertising to children, food retail strategies and pricing, and the impact of trade and investment agreements on national food environments [26].

In 2015, MANA supported an analysis of existing childhood obesity monitoring across the region (C-Pond, unpublished). This mapping revealed that, although several approaches are currently being utilised or developed by some countries (including the Global School-Based Student Health Survey for 13-17 year olds), childhood obesity data overall, and particularly for younger children (3-12 year olds), are lacking or underutilised. In PICTs where child body mass index (BMI) monitoring occurs, there is considerable variation in the methods used in schools and pre-schools (e.g. regular child health checks; variable age-targeted periodic or ad hoc BMI surveys) and few standardised protocols for measurement. The Review did not identify any countries that incorporate child anthropometric data in national health information systems or in national education information systems (C-POND, unpublished).

The issue of child obesity was discussed at the 2016 Heads of Health meeting and the need for a coordinated mechanism at regional level for cross country comparison was raised. MANA technical partners (particularly University of Hawai’i, C-POND, PIHOA and WHO) are collaborating for development of Pacific protocols for standardization of anthropometric measurement and to strengthen existing in-country BMI monitoring efforts to enable effective regional or national tracking of child weight trends and inform child obesity responses.

For the USAPI, rapid school and hybrid NCD and BRFSS (Behavioral Risk Factor Surveillance System) surveys being developed by PIHOA and CDC. These are designed to meet overlapping requirements of donors and, being easier and cheaper to deploy than their parent surveys, can be conducted more often.

### Accountability

**Fig. 2:**
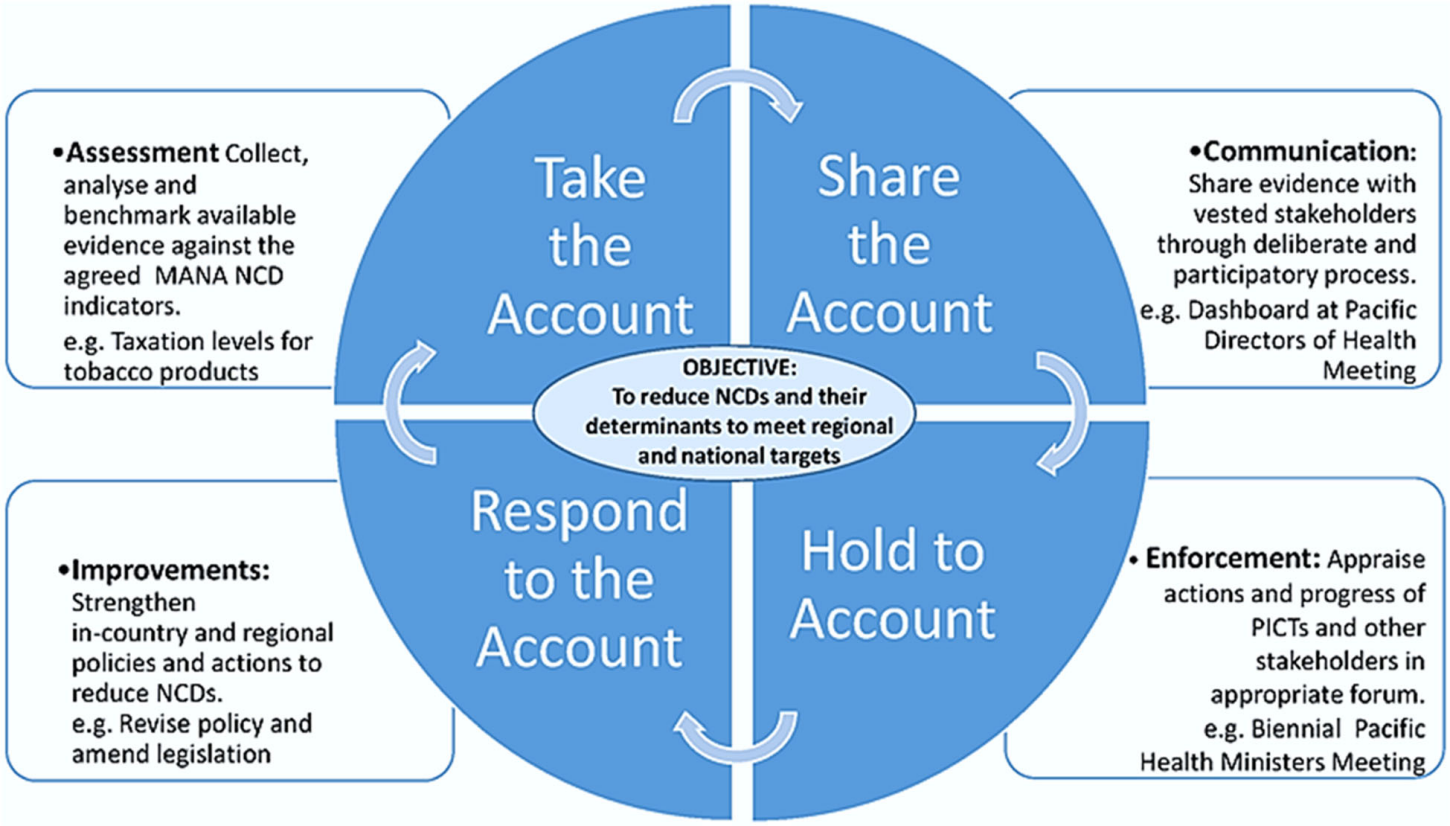
The Pacific MANA Accountability Framework (modified from Kraak et al. [27])

A significant achievement of MANA has been to create a shared interpretation across the partners of accountability as a core value in the framework, and to develop an appropriate mechanism to operationalise it. An accountability framework developed by Kraak and colleagues [27] was agreed as a valuable starting point at a MANA technical meeting in 2013, and was then adapted for the Pacific context (Fig. 2). Taking and sharing the account will be achieved through the development of the NCD Dashboard for Action (see next section). Holding to account will be achieved through the biennial Pacific Health Ministers meeting, attended by PICTs and key technical agencies. This meeting provides an opportunity for countries and agencies to be mutually accountable for action; i.e. space for specific actions or inactions to be openly discussed. Providing support for the fourth quadrant - ‘*responding to the account*’ - is a critical component of the framework for supporting partners to review, reassess or develop policies or actions for tackling specific issues.

MANA Partners (the blue section in Fig. 1) are supported by a multi-agency Coordination Team with an aim of achieving active, inclusive, and transparent lines of communication between the PICT-led Steering Committee and the alliance partners. The Coordination Team first formed in 2014 with representatives from C-POND, SPC, PIHOA, the University of Auckland and WHO. The composition of this team will continue to evolve as the alliance matures. Raising the profile of NCD monitoring as a holistic, complex, suite of critical and inter-related components highlights how active engagement with other existing networks e.g. PPHSN and the Brisbane Accord Group is critical to ensure that support for existing plans is maintained and efforts are not duplicated. For example, PPHSN has some similar structures/entry points through the Pacific Heads of Health meetings and improved harmonisation would be valuable. In addition, attention can be drawn towards other monitoring areas that receive less support or attention.

#### Development of a dashboard to help countries report on NCD action

Almost all PICTs have a five or ten year NCD strategy in place, including targets and indicators, which include numerous policy-based approaches. Most commonly these relate to taxation approaches for alcohol and tobacco; health-related food taxes; and settings-based policies [28]. Actions to increase import tariffs on specified “unhealthy” foods and lower tariffs on specified “healthy” foods in particular have increased since 2008 [29]. Most recently, policies for reducing consumption of sugar-sweetened beverages (SSB) have become a focus for the Pacific and half of the PICTs (12/22) now implement some form of raised tax on SSBs [30]. The Cook Islands, for example, have adopted the highest tax rate per kilo of sugar in SSB, while Tokelau has banned importation of carbonated sugar sweetened beverages since 2009. Despite these considerable actions, efforts to further strengthen policy commitment and implementation development are needed.

Dashboards are increasingly being used in many sectors as a means of visually presenting an organised profile of information [31, 32]. For NCDs, the dashboard for the CARICOM 2007 NCD Summit Declaration was one of the first [33]. In 2015, work began on a MANA Dashboard for Action which incorporates and expands on the set of indicators used for the WHO NCD Progress monitor 2015 [28]. Existing NCD dashboards focus predominantly on progress towards disease or risk factor targets.

The Dashboard for Action is focused on progress on implementing agreed policies and actions. Once finalised, the indicators included in the dashboard will provide a starting point for other countries/regions grappling with similar issues with accountability mechanisms for NCD action. The Dashboard is designed to be simple and flexible, yet have the rigour and credibility to serve as a national and regional mutual accountability mechanism. Moreover, alongside the related guidelines from the GMF, the WHO Western Pacific Regional Action Plan for the Prevention and Control of Noncommunicable Diseases (2014-2020) [34], the Pacific NCD Roadmap 2014, PIHOA’s NCD Emergency Response and NCD Policy Commitment Package and Toolkit [23] it serves to assist PICTs develop or revise their NCD strategies.

Ensuring that the information portrayed by the Dashboard is accurate and informative relies on clear and unambiguous criteria for which verifiable evidence can be collected. To avoid duplication, the Dashboard’s indicators and corresponding technical notes build on the ten process indicators developed by WHO for the 2015 NCD Progress Monitor [28]. To focus on action, the indicators cover four areas: governance (multi-sectoral taskforce, strategy); prevention policies (relating to tobacco, alcohol, food environments and physical activity); health system responses (access to NCD treatment and drugs, and tobacco cessation programs); and routine monitoring processes (adult and adolescent risk factor surveys and childhood body mass index).

**Fig. 3:**
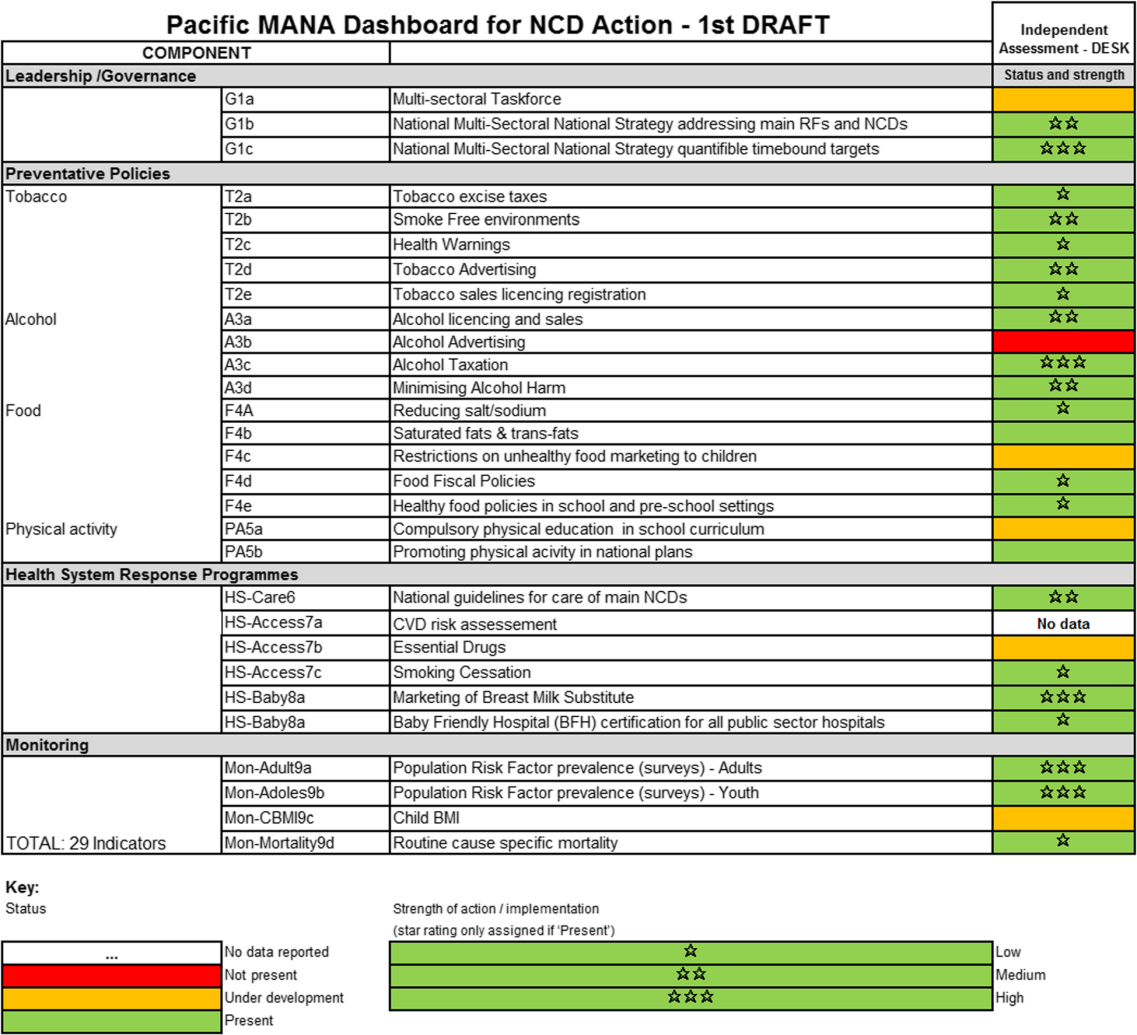
Draft Dashboard showing illustrative country status and strength and equivalent WHO Indicators (where relevant)

**Fig. 4:**
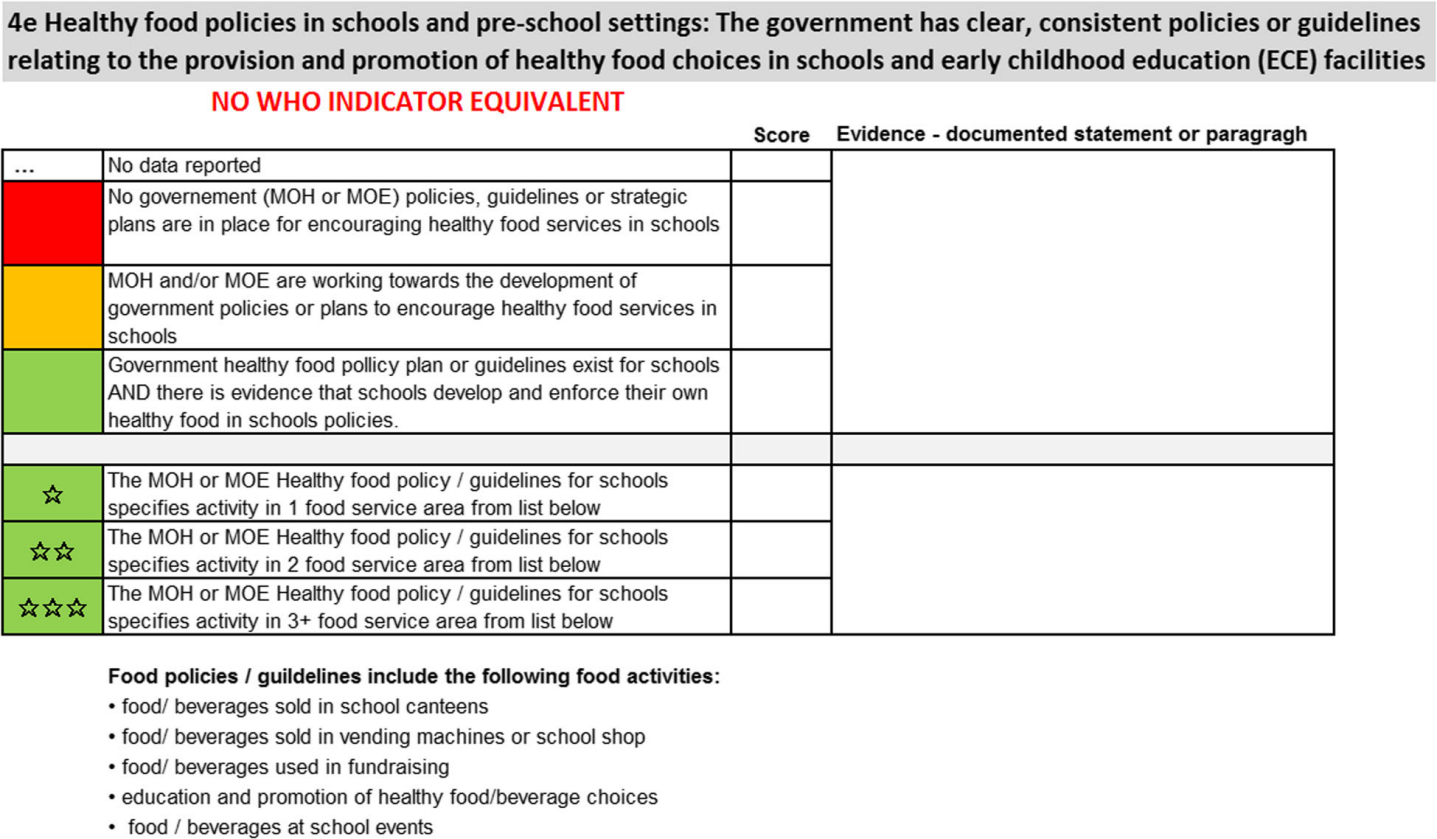
Example of technical notes for assessment of indicator F4e

For each indicator on the Dashboard, progress towards implementation of a policy or action is scored by a “traffic light” colour scheme: red for no policy present; amber for policy under development; and green for policy in place. For existing policies and actions (green light) the quality of the response, or degree of implementation, can be assessed against criteria provided in each indicator’s technical notes, refined through a one to three star system. Implicit in these notes is guidance for PICTs towards improvement. While the specific set of Pacific NCD indicators and assessment criteria remain under discussion, Fig. 3 provides a snapshot of indicators in the draft Dashboard. Figure 4 provides examples of the accompanying technical notes with assessment criteria for one of the indicators.

In a preliminary desk study trial it became clear that much of the data needed to populate the Dashboard are already stored in datasets within the various technical agencies or available online through country websites. To reduce the initial data collection burden on countries, it is envisaged that the MANA Coordination Team will, as far as possible, pre-populate the datasets before working with in-country contacts, to review the data, make amendments and fill the gaps. The completed dataset will be endorsed/verified by an appropriate country authority. Once the extant data have been entered, annual updating will be a simpler procedure for an in-country team.

There will inevitably be challenges in implementing the Dashboard nationally and regionally, not least will be sourcing verifiable country datasets and developing a sustainable storage and review mechanism for the database. It will be important to ensure that the storage mechanism is easy to access for data collection and updating, yet sufficiently sophisticated to allow interactive visualisations and national or cross-country reporting across multiple parameters. Setting up sustainable maintenance, reporting and updating mechanisms at the outset will be crucial for the Dashboard to become an effective mutual accountability tool for tracking NCD action.

Key to the success of the Dashboard will be a significant upfront commitment by all MANA partners in terms of time and an initial financial investment to establish a technologically robust, sustainable storage and reporting platform.

### Catalyzing change

Since its inception in 2014, MANA partners have catalysed two major changes in how NCDs and related actions are monitored in the Pacific; in particular:

#### Beyond business as usual

The collaboration of partners to formulate a common vision for accountability and a workable approach for monitoring and coordinating the myriad of NCD monitoring activities has been a valuable, if challenging, process. By seeing NCD monitoring as part of a complex, holistic process, the ‘business as usual’ approach, which has been rather siloed, is shifting towards an approach whereby organisational agendas are aligning and clarity is coming to roles and responsibilities. For example, the Coordination Team’s regular communication through virtual monthly meetings has allowed space for shared reporting of developments from around the region. Overall, the MANA framework has encouraged greater transparency, communication and shared understandings of mutual accountability between partners.

#### Accountability for policy action

Bring clarity to what ‘accountability’ actually means, and translating this into an assessment dashboard for actions on policies for the Pacific has been a major step forward. The methodology with detailed assessment notes received approval from the Pacific Heads of Health at their third meeting in February 2015 [35], and it was noted at the Forum Economics Ministers Meeting in October 2015 that, following the Eleventh Pacific Health Ministers Meeting in April [36], progress updates on the Dashboard for Action would be provided annually to Heads of Health, and at the biennial meetings of health and economics ministers, to introduce the mutual accountability mechanism in the region [37]. With a wider Healthy Islands indicator framework currently under development, the Pacific NCD Dashboard for Action will be able to underpin the NCD-related components of the assessment model for the *2015 Yanuca Island Declaration on Health in the Pacific Islands Countries and Areas*. The methodology has also been drawn upon to develop a “New tool … to help track progress in tobacco control” as reported in the January 2016 newsletter for Framework Convention Alliance - Pacific Island Countries [38].

## Summary/Conclusions

Creating multi-stakeholder systems for improving NCD monitoring for low-capacity countries across a region that is geographically vast, resource-constrained, *and* has the highest burden of NCDs in the world is challenging. It is anticipated that the formation of MANA as a collaborative monitoring alliance represents a major step forward for helping PICTs improve NCD actions. With consistent, sustained effort from countries and partners for maintaining the proposed NCD monitoring framework and mutual accountability mechanism, MANA has the potential to greatly support the Pacific in the translation of high level goals and targets into practical, relevant actions for the sustained reduction of NCDs.

This work to improve NCD monitoring in the Pacific will have important implications for other regions with resource constrained countries. As MANA moves forward, sharing the lessons learned in overcoming the technical, organisational, and political barriers to better NCD monitoring will be an important step in global collaborations to reduce NCDs.

## Abbreviations

CDC: US Centre for Disease Control and Prevention
CHL: Children’s Healthy Living Program for Remote Underserved Minority Populations in the Pacific Region
C-POND: Pacific Research Centre for Prevention of Obesity and NCDs
CRVS: Civil registration and vital statistics
GMF: Global Monitoring Framework
INFORMAS: International Network for Food and Obesity / non-communicable Diseases Research, Monitoring and Action Support
NCD: Non-communicable disease
PICTs: Pacific Island countries and territories
PIHOA: Pacific Islands Health Officers’ Association
POLHN: Pacific Open Learning Health Net
PPHSN: Pacific Public Health Surveillance Network
SHIP: Strengthening Health Information Program
SPC: Pacific Community
WHO: World Health Organisation

## Footnotes

1 The 12 countries with highest prevalence of diabetes are: American Samoa, Nauru, Cook Islands, Niue, Tokelau, Palau, Samoa, Tuvalu, the Federated States of Micronesia (f) / French Polynesia (m), Tonga, Kiribati, and Marshall Islands. Note: the country order varies with males and females (http://www.ncdrisc.org/dm-ranking-prevalence.html).

2 The 12 countries with highest obesity prevalence are: American Samoa, Cook Islands, Nauru, French Polynesia, Niue, Samoa, Palau, Tokelau, Tonga, Tuvalu, Marshall Islands, and the Federated States of Micronesia. Note: the country order varies with obese and severely obese, males and females (http://www.ncdrisc.org/ranking-prevalence-obesity.html).

3 There are a total of 22 Pacific Island countries and territories (PICTs). These are: American Samoa, Cook Islands, Federated States of Micronesia, Fiji, French Polynesia, Guam, Kiribati, Marshall Islands, Nauru, New Caledonia, Niue, Northern Mariana Islands, Palau, Papua New Guinea, Pitcairn Islands, Samoa, Solomon Islands, Tokelau, Tonga, Tuvalu, Vanuatu, and Wallis and Futuna,

4 Political commitments include: The WHO Framework Convention on Tobacco Control (FCTC), 2003; The Tonga Commitment, 2003; The Global Strategy on Diet Physical Activity and Health, 2004; The Pacific Framework for the Prevention and Control of Non-communicable Diseases, 2007; The Western Pacific Regional Action Plan for Non-communicable Diseases, 2009; The Global Strategy to Reduce the Harmful Use of Alcohol, 2010; The Honiara Communiqué, 2011; and The Apia Communiqué, 2013.

5 To maximise the effectiveness of efforts against NCDs, in 2007 SPC and WHO joined forces to develop a Pacific Framework for the Prevention and Control of NCDs and this formed the basis of the 2-1-22 Program - 2 organisations, 1 team and 22 countries.

6 INFORMAS (International Network for Food and Obesity / non-communicable Diseases Research, Monitoring and Action Support) is a global network of public-interest organisations and researchers that aims to monitor, benchmark and support public and private sector actions to create healthy food environments and reduce obesity and non-communicable diseases (NCDs) and their related inequalities. ^7^ The six northern United States affiliated Pacific Islands (USAPI) are: American Samoa, the Commonwealth of the Northern Mariana Islands, the Federated States of Micronesia, Guam, the Republic of Marshall Islands, and the Republic of Palau

7 The six northern United States affiliated Pacific Islands (USAPI) are: American Samoa, the Commonwealth of the Northern Mariana Islands, the Federated States of Micronesia, Guam, the Republic of Marshall Islands, and the Republic of Palau
